# Heterogeneity and regulation of oligodendrocyte morphology

**DOI:** 10.3389/fcell.2022.1030486

**Published:** 2022-10-24

**Authors:** Yasuyuki Osanai, Reiji Yamazaki, Yoshiaki Shinohara, Nobuhiko Ohno

**Affiliations:** ^1^ Department of Anatomy, Division of Histology and Cell Biology, School of Medicine, Jichi Medical University, Shimotsuke, Japan; ^2^ Australian Regenerative Medicine Institute, Monash University, Clayton, VIC, Australia; ^3^ Department of Anatomy and Cell Biology, Faculty of Medicine, University of Yamanashi, Chuo, Japan; ^4^ Division of Ultrastructural Research, National Institute for Physiological Sciences, Okazaki, Japan

**Keywords:** oligodendrocyte, myelin, internode, selectivity, conduction velocity, neuronal activity, remyelination

## Abstract

Oligodendrocytes form multiple myelin sheaths in the central nervous system (CNS), which increase nerve conduction velocity and are necessary for basic and higher brain functions such as sensory function, motor control, and learning. Structures of the myelin sheath such as myelin internodal length and myelin thickness regulate nerve conduction. Various parts of the central nervous system exhibit different myelin structures and oligodendrocyte morphologies. Recent studies supported that oligodendrocytes are a heterogenous population of cells and myelin sheaths formed by some oligodendrocytes can be biased to particular groups of axons, and myelin structures are dynamically modulated in certain classes of neurons by specific experiences. Structures of oligodendrocyte/myelin are also affected in pathological conditions such as demyelinating and neuropsychiatric disorders. This review summarizes our understanding of heterogeneity and regulation of oligodendrocyte morphology concerning central nervous system regions, neuronal classes, experiences, diseases, and how oligodendrocytes are optimized to execute central nervous system functions.

## Introduction

Understanding the information processing in the central nervous system (CNS) by exploring brain structures has been a challenge in life science. Evidence demonstrates that the regulation of nerve conduction by myelin ensheathment plays a critical role in the development, physiology, and diseases of CNS. The term myelin was coined by Rudolf Ludwig Virchow (1821–1902), but at that time, myelin was thought to be produced by neurons (Boullerne, 2016). Pio del Río Hortega showed that oligodendrocytes form intimate interactions with the neuronal axons in CNS (Rio Hortega, 1928; Perez-Cerda et al., 2015), followed by observation of multilamellar myelin ensheathment using ultrastructural analyses in the 1940s and 50s (Rosenbluth, 1999). Myelin sheaths increase the conduction velocity of neuronal axons by 50–100 times by reducing the axonal membrane’s capacitance and enabling saltatory conduction (Tasaki, 1939). While myelin formation has been conventionally viewed as developmentally determined, stereotyped, and rather rigid after formation, recent studies have revealed that the regulation of myelin ensheathment is dynamic and essential for basic and higher brain functions and is deeply involved in disease pathophysiology. Understanding the regulatory mechanisms of myelin formation, maintenance, and plasticity by oligodendrocytes in the CNS would significantly contribute to the knowledge about the brain functions and diseases.

Oligodendrocytes form multiple myelin sheathes on neuronal axons in the CNS, in contrast to Schwann cells in the peripheral nervous system, where a single Schwann cell forms myelin sheath on a single axon. This unique characteristic of oligodendrocytes raises the question of which axons are myelinated by individual oligodendrocytes. Recent tools such as transgenic mice, viral vectors, two-photon microscopy, and three-dimensional analyses with electron microscopy (3D-EM) have enabled us to observe dynamic and complex interactions between oligodendrocytes and other cells and reveal patterns of myelination in a particular class of neuronal axons. Studies revealed that the oligodendrocytes dynamically change their morphology depending on experience or diseases, and the regulation of myelination can be biased toward a particular set of axons. Recent studies revealed that oligodendrocytes have heterogeneous morphology and gene expression patterns in different CNS regions, suggesting that the morphology of oligodendrocytes is optimized to execute the function of each CNS region. In this review, we summarized oligodendrocyte/myelin morphological changes depending on neuronal classes, experience, and diseases and discussed their connection to brain functions.

## Oligodendrocyte morphology and its contribution to neural information processing

The characteristic morphology of individual oligodendrocytes is established through sequential events of migration and differentiation of oligodendrocytes characterized by the expression of specific markers. The oligodendrocyte progenitor cells (OPC) positive for NG2 and PDGFRa originate from specialized areas of the subventricular zones and migrate with bipolar morphology to distribute throughout the CNS regions (Bergles and Richardson, 2015). The OPC generate processes and differentiate into pre-myelinating oligodendrocytes positive for markers such as O4 and proteolipid protein (PLP) after proliferation and migration. While many of these early oligodendrocyte lineage cells degenerate and are lost during development (Hughes and Stockton, 2021), surviving cells differentiate into myelinating and matured oligodendrocytes expressing markers such as myelin basic protein (MBP) and myelin-associated oligodendrocytic basic protein (MOBP), and produce myelin sheathes around multiple axons localized at the vicinity of their extended processes. The extension of process could be primarily limited but not restricted to a small range; oligodendrocytes do not myelinate axons closest to cell body, as observed with intrafascicular oligodendrocytes in the white matter (Tanaka et al., 2021).

**FIGURE 1:**
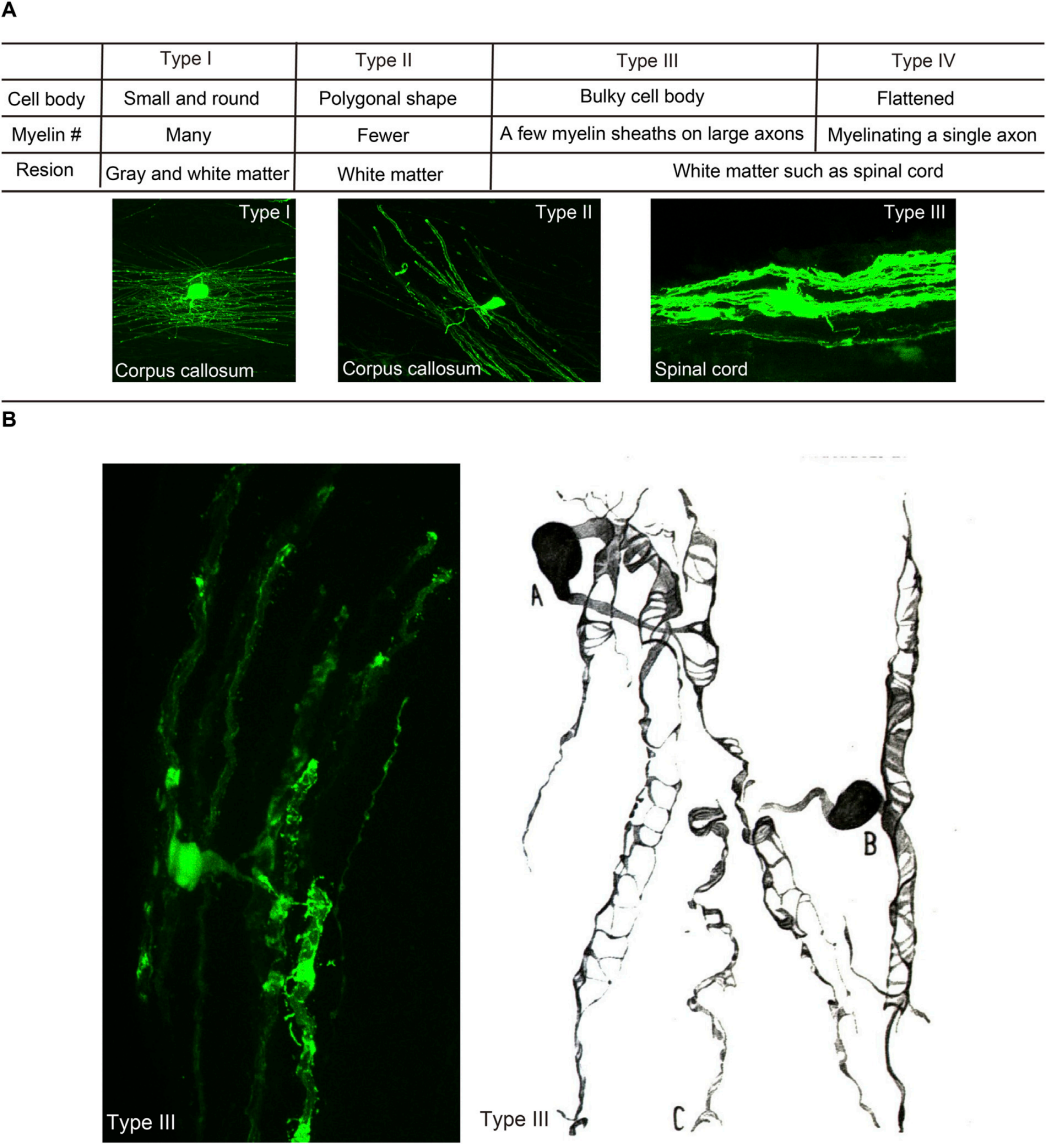
Morphological heterogeneity of oligodendrocytes. **(A)** Characteristics and shapes of different types of oligodendrocytes, following the classification by Pio del Río Hortega (1928). **(B)** Type III oligodendrocyte in the mouse spinal cord (Osanai et al., 2017) and that in the cat medulla oblongata described by Rio Hortega (1928).

The mature oligodendrocytes are frequently shown as cells with small cell bodies and myelin sheathes at the tips of processes. However, the morphology of individual oligodendrocytes and their myelin could be variable in different brain regions depending on the structures of ensheathed axons. When oligodendrocytes were first discovered, they were classified into four types (Figure 1). Type I oligodendrocytes have a small round shape cell body and many processes and are found in gray and white matter. Type II oligodendrocytes possess a polygonal shaped cell body with fewer processes and are found only in the white matter. Type III oligodendrocytes have a bulky cell body forming a few myelin sheaths on large caliber axons in the white matter such as the spinal cord. Type IV oligodendrocytes, also called Schwannoid oligodendrocytes, myelinate a single large caliber axon similar to Schwann cells (Rio Hortega, 1928; Perez-Cerda et al., 2015) in the white matter of the brain stem or spinal cord. The structural variance of individual oligodendrocytes is widely recognized, and thus the number of ensheathments by each oligodendrocyte is regulated by the sizes of ensheathed axons. Oligodendrocytes myelinating thin axons have more myelin ensheathments (Fanarraga et al., 1998). Oligodendrocytes tend to form longer and thicker myelin on axons or fibers with larger diameters, and axonal diameters could also be modulated by myelin ensheathments (Hildebrand et al., 1993). Therefore, structural properties between oligodendrocytes and axons are considered to be determined through their reciprocal interactions.

**FIGURE 2:**
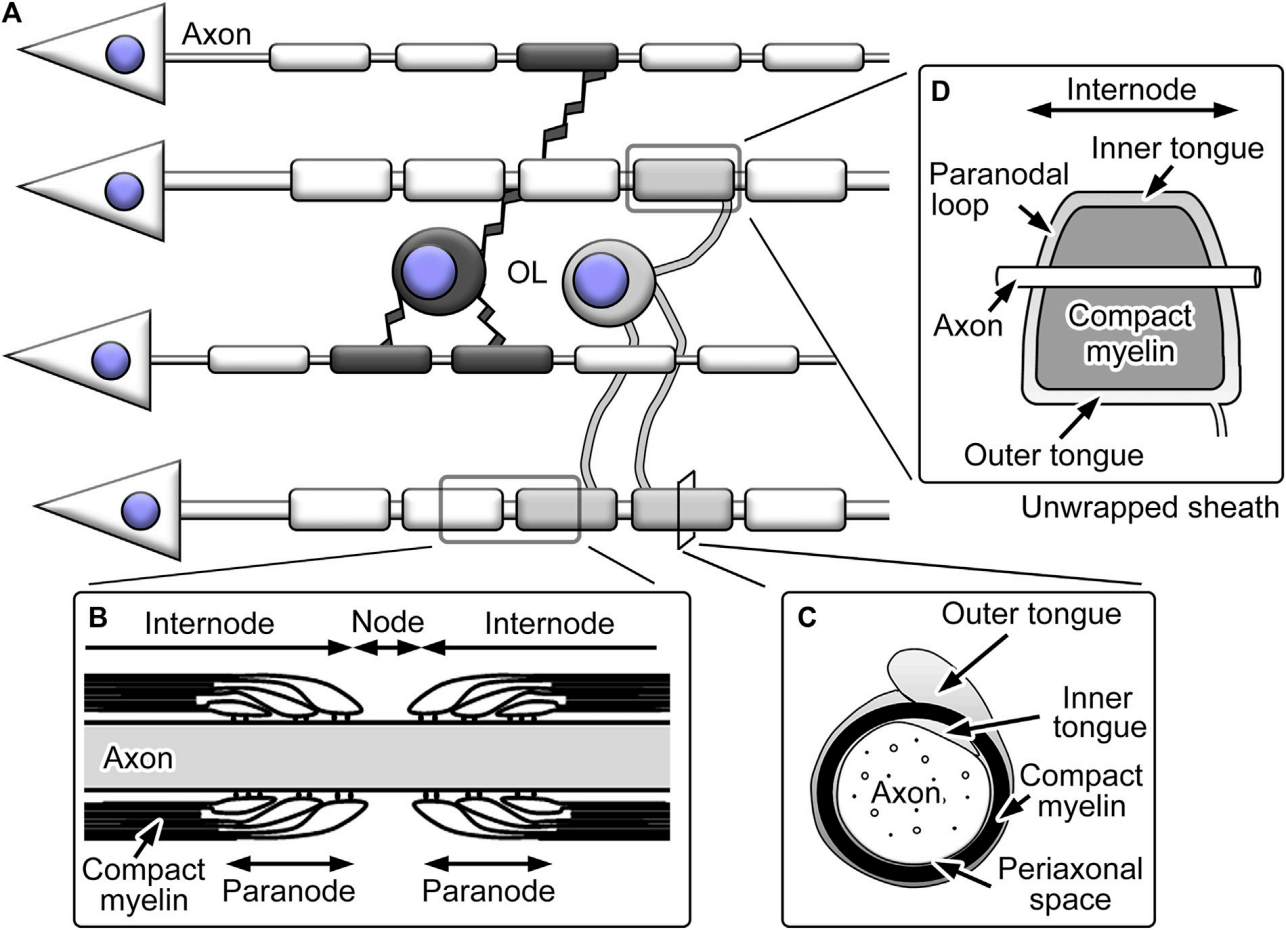
Schemes showing structures of oligodendrocytes (OL) and their myelin. **(A)** OL typically extend multiple processes and form myelin ensheathment around neuronal axons. **(B)** The axons with myelin ensheathment can be divided into nodes of Ranvier and internodes. The nodes are flanked by paranodes with paranodal loops and transverse bands. **(C)** The internodal myelin sheaths include compact myelin with inner and outer tongues of cytoplasmic channels, and the periaxonal space represents the space between axolemma and adaxonal membranes of oligodendrocytes. **(D)** When the internodal myelin sheaths are unwrapped, the inner and outer tongue processes; the latter is connected to the process from the cell body, continues throughout the internode and is connected with the paranodal loops.

The myelin ensheathment by oligodendrocytes divides axons into domains with distinct morphology, functions, and molecular distribution (Figure 2). The large part of single myelinated fibers is internode and largely covered by the tightly packed spiral plasma membranes termed compact myelin. The compact myelin accompanies cytoplasmic channels called inner and outer tongues at the inner and outer surfaces of compact myelin, respectively, both longitudinally continuing in the entire internode and the latter connected to processes originating from cell bodies. The gaps between internodal myelin ensheathment are the nodes of Ranvier. These nodal regions are important for rapid saltatory conduction, as the high concentration of Na^+^ channels at the nodes mediates local depolarization (Waxman, 2006). The regions flanking the nodes are called paranodes and include paranodal cytoplasmic channels connected to inner and outer tongues (Peters et al., 1991). Paranodal cytoplasmic channels form paranodal loops, which juxtapose closely to axonal cell membranes. The electron-dense transverse bands between the membranes represent the junctional complexes between myelin and axons, including multiple membranous and membrane cytoskeletal proteins (Poliak and Peles, 2003). The axo-glial junctions in the paranodes are called septate-like junctions and function as a diffusion barrier for small molecules (Rosenbluth, 2009).

The conduction velocity of myelinated axons is regulated by their structural parameters, such as fiber diameter, myelin thickness, and internodal length (Waxman, 1980). The rapid saltatory conduction is achieved by inhibiting ion exchange by internodal myelin sheathes with high transverse resistance (Ritchie, 1995). The conduction velocity is highly dependent on axonal and fiber (axon and myelin) diameter, but it dependents on myelin thickness when the axon diameter is fixed. The g-ratio is calculated as the ratio of axonal and fiber diameters. It has been proposed that the g-ratio should have an optimal value of around 0.6–0.7 to maximize conduction velocity (Smith and Koles, 1970). A particular internode distance might be important to achieve a maximum conduction velocity at any given diameter (Brill et al., 1977). The length of the nodes and the size of the periaxonal space are modified within active brain regions, which modulate nerve conduction; enlarged nodal length and periaxonal space decrease the velocity (Richardson et al., 2000; Etxeberria et al., 2016; Arancibia-Carcamo et al., 2017; Cullen et al., 2021). The paranodal diffusion and associated structures could also contribute to the regulation of conduction velocity, and impaired septate-like junctions slow nerve conduction (Bhat et al., 2001; Sherman et al., 2005; Yamazaki et al., 2010).

While the structures of oligodendrocytes and their myelin are critical in the modulation of nerve conductions of neural circuits, it has been challenging to understand the morphology of individual oligodendrocytes in CNS. Multiple analytical methods have been developed to elucidate different aspects of the structures of oligodendrocytes and myelin and to reveal the substantial heterogeneity of oligodendrocytes. The next section is focused on the methods used for analyzing individual oligodendrocytes and their myelin.

## Methods for observing individual oligodendrocyte morphology

The morphology of individual oligodendrocytes in the superficial layers of the cortex can be observed using conventional methods. However, due to the high density of oligodendrocytes and their myelin, it is challenging to visualize individual oligodendrocyte morphology in the deep layers of cortex and white matter using immunostaining methods. The silver carbonate method is the classical method of observing individual oligodendrocyte morphology, which can produce clear oligodendrocyte images. But the method requires special fixation, and results are variable (McCarter, 1940). The intracellular injection of dye is a powerful method for labeling a targeted single oligodendrocyte and can be used after single-cell electrophysiology (Weruaga-Prieto et al., 1996; Yamazaki et al., 2007). However, the problem of this method is the difficulty of increasing the sample number, and the population of labeled oligodendrocytes can be affected by the experimenter’s bias.

The most commonly used method for analyzing single oligodendrocyte morphology is labeling with fluorescent proteins. An example is Tau-mGFP mice which possess lox-STOP-lox-mGFP-IRES-NLS-LacZ transgene and express mGFP and nucleus-targeted LacZ in a Cre-dependent manner under control of Tau promoter that is active in neurons and mature oligodendrocytes (Hippenmeyer et al., 2005; Young et al., 2013; Tripathi et al., 2017). These mice can be crossed with mice expressing Cre in OPC, such as PdgfRa-CreERT2 (Rivers et al., 2008) and NG2-CreERT2 mice (Huang et al., 2014), to label newly generated oligodendrocytes that are differentiated after tamoxifen administration. Since mature oligodendrocytes express opalin and PLP, opalin-CreERT2 mice (Tripathi et al., 2017) and PLP1-CreERT mice (Jeffries et al., 2016) can be crossed with the Tau-mGFP mice for labeling preexisting mature oligodendrocytes that are differentiated before tamoxifen administration. Another transgenic mouse line useful for analyzing single oligodendrocyte morphology is MBP-maEGFP mice, where less than 1% of oligodendrocytes express membrane-associated EGFP. Therefore, individual oligodendrocytes and their fine processes are sparsely labeled and are clearly visualized. The drawback of employing mice is the difficulty in predicting the brain region where oligodendrocytes are predominantly labeled (Chong et al., 2012). For observing living oligodendrocytes by two-photon microscopy, PLP-EGFP mouse (Mallon et al., 2002; Griemsmann et al., 2015; Gould et al., 2018; Snaidero et al., 2020) or MOBP-EGFP mouse (Gong et al., 2003; Hughes et al., 2018; Larson et al., 2018; Orthmann-Murphy et al., 2020) are widely used. These transgenic mice used for two-photon microscopy studies are suitable for observing shallow layers of oligodendrocytes. However, observing oligodendrocytes in deep layers and the white matter is difficult because oligodendrocytes are densely labeled in the regions. A recent study using MOBP-EGFP mice and two-photon microscopy found that motor learning increased the proportion of myelin sheath retraction (Bacmeister et al., 2020).

Another way to label oligodendrocytes fluorescently is postnatal gene transduction, such as the usage of viral vectors. While adeno-associated virus (AAV) vectors are widely used to label neurons, it is difficult to observe oligodendrocyte morphology using AAV vectors *in vivo*, because AAV densely labels oligodendrocytes and other cell types at the injected sites. In contrast, stereotaxic injection of attenuated rabies virus vectors or Semliki Forest virus vectors sparsely labels oligodendrocytes and enables observing the morphology of individual oligodendrocytes in the white matter (Osanai et al., 2017; Toth et al., 2021). The advantage of using viral vectors for labeling oligodendrocytes is that oligodendrocytes in the targeted white matter are reliably labeled. We previously used an attenuated rabies virus vector to observe oligodendrocyte morphology in the mouse corpus callosum and AAV vectors for labeling neuronal axons from motor and sensory cortices. We found oligodendrocytes in the mouse corpus callosum preferentially myelinated axons from the sensory cortex (Osanai et al., 2017).

The development of electron microscopy in the mid-20th century unequivocally identified the myelin formation around the axons by oligodendrocytes. It revealed the ultrastructure of nerve fibers with myelin ensheathment (Rosenbluth, 1999). Observation with electron microscopy is still widely used and essential to reveal detailed ultrastructure of oligodendrocytes and their myelin in various animals, including humans (Trapp and Kidd, 2004). Tri-dimensional ultrastructural analyses using serial electron microscopic images are the powerful for analyzing the morphology of neurons and glia, including oligodendrocytes close to the whole cell level and their ultrastructure (Briggman and Bock, 2012; Ohno et al., 2015). Various subtypes of neuronal axons with different axonal diameters are intermingled in the gray and white matter. Axons with diameters greater than 400 nm can be myelinated (Lee et al., 2013); while some axons are unmyelinated even if the diameters are larger than the threshold (Sturrock 1980). Interestingly, recent studies suggested that a portion of oligodendrocytes preferentially myelinate inhibitory interneurons, and a single oligodendrocyte myelinates axons of similar diameter with similar myelin thickness (Zonouzi et al., 2019; Tanaka et al., 2021). In addition to immunohistochemical methods to label specific molecules and cells in electron microscopic images (Katoh et al., 2017), genetically-encoded probes for specific labeling in electron microscopy have been developed (Shu et al., 2011; Zhang et al., 2019). For example, dAPEX2 is an artificially modified peroxidase optimized for visualization in electron microscopic analyses. It can be targeted to specific cellular compartments and detected by reaction with hydrogen peroxide and diaminobenzidine (Zhang et al., 2019). Expressing dAPEX2 in different compartments, such as mitochondria and endoplasmic reticulum, in various types of cells enables us to distinguish the ultrastructure of those differentially labeled cells in electron microscopic analysis. Such electron microscopic advancement can also reveal the heterogeneity of oligodendrocytes which potentially associated with specific neural circuits and CNS functions under physiological and pathological conditions.

Using these approaches for observing individual oligodendrocytes, whose advantages and disadvantages are summarized in Table 1, previous studies revealed substantial heterogeneity of oligodendrocyte morphology in the CNS. In the next section, the current understanding of the morphological heterogeneity of oligodendrocytes is reviewed.

**TABLE 1:** The characteristics of the methods for observation of individual oligodendrocytes.

Methods for single oligodendrocyte labeling	Advantages	Disadvantages	Ref
Silver carbonate method	Clear oligodendrocyte image	Special fixation required	Rio Hortega, 1928
	Permanent preparation	Variable results	McCarter, (1940)
Intracellular injection of dye	Clear oligodendrocyte image	Low sample number	Weruaga-Prieto et al. (1996)
	Can be used after single cell electrophysiology	Selection bias for single cell dye injection	Yamazaki et al. (2007)
Tau-mGFP mice x OPC- or Oligodendrocyte-Cre mice	Reliably labels oligodendrocytes	Immunostaining is necessary	Young et al. (2013)
	Easy to obtain the transgenic mouse from the Jackson laboratory	Need to cross breed with Cre lines	Tripathi et al. (2017)
	Labeling oligodendrocytes generated after or before tamoxifen injection	Loss of *Tau* exon2 in one allele	Pan et al., 2020
MBP-maEGFP mice	Sparsely labels oligodendrocytes in multiple CNS regions	Immunostaining is necessary	Chong et al. (2012)
	No crossbreading with Cre lines required	Unpredictable which regions of oligodendrocyte will be labeled	
Oligodendrocyte promoter-XFP mice such as PLP-EGFP	Suitable for two-photon microscopic analysis	Individual oligodendrocyte morphology can be accessed only in shallow cortical layers	Mallon et al. (2002)
	All oligodendrocytes will be labeled		Hughes et al. (2018)
Attenuate rabies virus or semliki forest virus injection	Very clear oligodendrocyte image	Legal use restrictions	Osanai et al. (2017)
	Immunostaining is unnecessary	Cytotoxicity	Osanai et al. (2018)
	No transgenic animals required	Only for white matter oligodendrocytes	Toth et al. (2021)
3D-EM	Highest resolution of oligodendrocyte image	Laborious and time-consuming	Briggman and Bock, (2012)
	Fine structures such as myelin thickness can be observed	Low sample number	Zonouzi et al. (2019)
	Interaction with other celltypes can be observed	Needs expert help	Tanaka et al. (2021)

## Heterogeneity of oligodendrocyte morphology among central nervous system regions

Individual CNS regions possess different functions and different populations of neurons, and oligodendrocyte population also appears to differ among CNS regions. We summarized the morphological feature of myelinating oligodendrocytes in each CNS region (Table 2). The mean number of myelin sheathes produced by individual oligodendrocytes is the highest, and their mean internodal length is the shortest in the cerebral cortex compared to the other CNS regions. Oligodendrocytes in the corpus callosum possess a lower number of myelin sheaths and longer sheaths than those in the cerebral cortex, although many axons are derived from cerebral cortex in both regions (mean # of myelin sheath, cortex 34, corpus callosum 23, mean internodal length; cortex 53 µm and corpus callosum 96 µm) (Chong et al., 2012; Osanai et al., 2017; Bacmeister et al., 2020). It is also possible that the oligodendrocytes present in different layers have differential morphology and functions and a variable distribution of myelin internodes in different layers of the cerebral cortex within a single axon (Tomassy et al., 2014). The average internodal length of the myelin sheath is the longest in the spinal cord white matter (average 385 µm) (Lasiene et al., 2009). Myelin sheaths appear to be longer in the regions containing long projection axons, such as the optic chiasm (average 191 µm) (Osanai et al., 2022) and spinal cord (average 385 µm). The formation of the longer myelin sheath around long-distance projecting axons seems reasonable to transmit neural information quickly. In a recent study utilizing artificial axon-like microfiber, OPCs obtained from the cerebral cortex formed shorter myelin sheath than OPCs from the spinal cord *in vitro*, indicating that OPCs in the cerebral cortex and spinal cord intrinsically form different myelin sheathes following differentiation (Bechler et al., 2015; Bechler et al., 2018). On the other hand, the microenvironment determined by neuronal axons, such as neuronal activity and GABA, also impacts oligodendrocyte morphology (Hamilton et al., 2017; Mitew et al., 2018; Osanai et al., 2018). Thus, morphological differences of oligodendrocytes among CNS regions seem to be shaped by endogenous and environmental factors.

**TABLE 2:** The number of myelin sheaths per oligodendrocyte and myelin internodal length in each CNS region.

	# Of myelin sheaths formed per OL		Internodal length	
CNS region	(Range, Average)	Ref	(Range, Average)	Ref
Cerebral cortex	(23–58, 34)	Chong et al. (2012)	(15–103 µm, 53 µm)	Bacmeister et al. (2020)
Corpus callosum	(15–32, 23)	Osanai et al. (2017)	(18–196 µm, 96 µm)	Chong et al. (2012)
Caudate putamen	(12–34, 22)	Chong et al. (2012)	(6–238 µm, 100 µm)	Chong et al. (2012)
Cerebellum white matter	(2–7, 5)	Toth et al. (2021)	(38–169 µm, 84 µm)	Toth et al. (2021)
Spinal cord white matter	(3–40, 10)	Osanai et al. (2017)	(<100–600< µm, 385 µm)	Lasiene et al. (2009)
Optic chiasm	(5–30, 17)	Osanai et al. (2017)	(67–362 μm, 191 µm)	Osanai et al. (2022)

Some classes of oligodendrocytes are found only in certain brain regions. In the gray matter, satellite oligodendrocytes are closely located to the neuronal Soma, but their functions are not fully understood (Baumann and Pham-Dinh, 2001; Battefeld et al., 2016). In the white matter such as corpus callosum and optic nerve, oligodendrocytes tend to form bead-like clusters in rows easily observed by nuclear staining. The mechanism of forming the row cell clusters and their functions are unclear. We recently found that individual oligodendrocytes in the row myelinate distinct axons; in other words, oligodendrocytes in the row are heterogenous in the axonal selection and myelin formation (Tanaka et al., 2021). Future studies should address the mechanisms of row formation and intercellular communication of oligodendrocytes in the row.

Single-cell RNA sequencing (scRNA-seq) of oligodendrocytes in multiple CNS regions revealed that the ratio of oligodendrocyte subtypes varies within CNS regions (Marques et al., 2016). Reports indicate that some oligodendrocyte subtypes defined by scRNA-seq are localized in a particular CNS region, and their functions are different (Marques et al., 2016; Floriddia et al., 2020; Marisca et al., 2020), indicating that the heterogeneity of oligodendrocyte gene expression is linked to location of oligodendrocytes in the brain and is likely correlated to oligodendrocyte morphology. The scRNA-seq analysis indicated that particular subtypes of mature oligodendrocytes express synaptic proteins, suggesting that some mature oligodendrocytes can monitor neuronal activity (Marques et al., 2016). Studies revealed the presence of synapse-like structure under myelin sheath in a portion of myelin sheathes (Proctor et al., 2015; Hughes and Appel, 2019). Oligodendrocyte morphology and gene expression vary among CNS regions, and future studies should address the mechanisms of morphogenesis and functions of the oligodendrocyte subtypes.

## Mechanism regulating oligodendrocyte morphology

Recent studies have revealed the interaction between neuronal activity, myelination, and oligodendrocyte morphology (Bonetto et al., 2021). Increasing neuronal activity of the motor cortex resulted in an increasing number of mature oligodendrocytes, increased myelin thickness in the brain, and improved motor function in mice (Gibson et al., 2014). However, how myelination is controlled by neuronal activity or experience is still under debate. In the developing spinal cord, myelination of neuronal axons is not prevented by a sodium channel blocker tetrodotoxin in juvenile zebrafish (Hines et al., 2015). The percentage of myelinated axons of retinal ganglion cells (RGCs) is unaffected by visual deprivation, given that almost all RGC axons are myelinated in animals with dark rearing or monocular deprivation (Moore et al., 1976; Etxeberria et al., 2016; Osanai et al., 2022), indicating that the impact of reducing neuronal activity on the number of myelinated axons in the spinal cord and optic nerve are minimum. However, recent studies on oligodendrocyte morphology at the single-cell level revealed that visual deprivation significantly altered myelin internodal length (Etxeberria et al., 2016; Osanai et al., 2018; Osanai et al., 2022). In the auditory system, reduced auditory stimulation by earplugs reduces the myelin thickness of trapezoid body fibers, which are important for sound localization (Sinclair et al., 2017). The nascent myelin sheath length formed on developing zebrafish spinal cord is reduced by inhibiting synaptic vesicle exocytosis of a particular class of axons (Hines et al., 2015). We also found that reduced sensory stimuli decrease myelin internodal length in optic chiasm and corpus callosum using monocular deprivation and whisker removal models, respectively (Osanai et al., 2018). Thus, decreased myelin sheath length at the single-cell level seems to be a general response to decreased neuronal activity in multiple CNS regions for mice and zebrafish (Hines et al., 2015; Koudelka et al., 2016; Sinclair et al., 2017; Osanai et al., 2018; Osanai et al., 2022). It is unclear why broad inhibition of neuronal activity by tetrodotoxin does not affect myelination while inhibition of a particular class of neuronal axons reduces myelin sheath length of zebrafish spinal cord (Hines et al., 2015).

Neuronal activity’s effects on myelination seem to differ between CNS regions. Increased activity of callosal projecting neurons increased the number of mature oligodendrocytes and increased myelin thickness in the mouse corpus callosum (Gibson et al., 2014; Mitew et al., 2018). In contrast, decreased visual stimuli increased the number of mature oligodendrocytes and thickened myelin sheaths in the mouse optic nerve (Etxeberria et al., 2016; Mayoral et al., 2018; Osanai et al., 2022). Differences in myelination responses to neuronal activity and stimuli may reflect functional and structural differences between the optic nerve and corpus callosum. A recent report indicated that visual deprivation modulates myelination of a specific subtype of neuron, parvalbumin-expressing GABAergic interneuron in the mouse visual cortex (Yang et al., 2020), suggesting an impact of neuronal activity or experience can be different between CNS regions and neuronal subtypes.

It was revealed in previous studies that new oligodendrocytes are generated in response to the experience and that oligodendrocyte generation is required for motor learning and remote memory (Mckenzie et al., 2014; Xiao et al., 2016; Pan et al., 2020; Steadman et al., 2020). However, the extent of difference between the newly generated and pre existing oligodendrocyte morphology is unclear. Some oligodendrocytes preferentially myelinate a particular subtype of neurons (Osanai et al., 2017; Zonouzi et al., 2019; Tanaka et al., 2021). The pharmacological increase in activity of the axonal subset could enhance oligodendrocyte generation and the formation of thicker myelin toward the axons with increase in their activity (Mitew et al., 2018). An intriguing possibility is that the newly generated oligodendrocytes, upon behavioral changes, preferentially myelinate axons in the neuronal circuit responsible for the behavior and has a unique morphology with a particular physiological function. In future studies, it will be necessary to establish the morphological heterogeneity of newly generated and pre-existing oligodendrocytes and their myelin under different behavioral tasks to address this possibility.

Social isolation results in behavioral dysfunction with abnormal oligodendrocyte/myelin morphology in the prefrontal cortex (PFC) (Liu et al., 2012; Makinodan et al., 2012). In a seminal study of social isolation, mice reared in isolated conditions during P21 to P35 had significant decrease in the myelin thickness and branch points of oligodendrocyte processes along with decreased expression of ErbB3 ligand neuregulin-1 in the PFC (Makinodan et al., 2012). In adult mice, longer-term social isolation (8 weeks) induced behavioral changes and myelin ultrastructural changes in the PFC (Liu et al., 2012). It was also suggested in the studies that decreased stimulation did not affect the number of mature oligodendrocytes whereas it altered morphology of oligodendrocyte/myelin, specifically in the PFC. Increasing myelination by clemastine administration rescues behavioral dysfunction in socially isolated mice (Liu et al., 2016). Swire et al. (2019) indicated that a vasoactive peptide endothelin expression is reduced in the PFC of socially isolated mice, and the administration of endothelin receptor B agonist normalized the morphology of oligodendrocyte/myelin of socially isolated mice (Swire et al., 2019). This suggests that adaptive myelination is partly regulated by blood flow.

In addition to neuronal activity, microglia and astrocyte have been implicated as environmental factors regulating oligodendrocyte morphology. Microglia regulate oligodendrocyte number and morphology through myelin pruning and phagocytosis of OPC (Hughes and Appel, 2020; Nemes-Baran et al., 2020; Djannatian et al., 2021). Astrocytes secrete leukemia inhibitory factor-like protein (LIF) and thrombin protease inhibitors that regulate myelination and myelin sheath thickness (Gard et al., 1995; Ishibashi et al., 2006; Dutta et al., 2018). Astrocytes can regulate myelination through gap junctions between oligodendrocyte and astrocytes (Orthmann-Murphy et al., 2008; Lutz et al., 2009; Domingues et al., 2016).

Recent studies have also revealed the intrinsic molecular mechanism modulating oligodendrocyte morphology. Tubulin polymerization promoting protein (TPPP) that regulates myelin sheath number, myelin thickness, and myelin internodal length formed by oligodendrocytes (Fu et al., 2019), and hyperpolarisation-activated cyclic nucleotide-gated (HCN) channel2 regulates myelin internodal length (Swire et al., 2021). Receptors of neurotrophic factors or neurotransmitters are involved in regulating oligodendrocyte/myelin morphology; myelin thickness is reduced when the BDNF receptor, TrkB, is removed in oligodendrocytes (Wong et al., 2013; Geraghty et al., 2019). For proper myelination, physical properties of the microenvironment, such as stiffness of extracellular matrix or axonal diameter, are important (Lee et al., 2013; Segel et al., 2019). The physical properties of the microenvironment could be sensed by mechano-sensors such as YAP regulates oligodendrocyte process number (Shimizu et al., 2017; Shimizu et al., 2019). Cell adhesion molecules such as N-Cadherin (Miyata et al., 2011; Chen et al., 2017), laminin (Kang and Yao, 2022), CADM1 (Hughes and Appel, 2019), CADM4 (Elazar et al., 2019), neuroligin (Proctor et al., 2015) are necessary for proper morphology of oligodendrocytes (Colognato and Tzvetanova, 2011). Teneurin-4, which could be associated with Fyn and focal adhesion kinase, has been proposed to regulate formation of oligodendrocyte processes and myelination of small-diameter axons (Hoshina et al., 2007; Suzuki et al., 2012; Hayashi et al., 2020).

Collectively, oligodendrocyte number and morphology are regulated by intrinsic and extrinsic factors, while the impact of that varies with population of activity-modulated neuronal axons, developmental timing, and CNS regions. On the other hand, their morphology is substantially changed under pathological conditions, and such changes are implicated in the pathophysiology of neurological diseases.

## Pathological changes of oligodendrocyte morphology

Morphological abnormalities in oligodendrocytes are likely involved in various pathological conditions, given that such morphogenesis is important during myelination and remyelination. Multiple sclerosis (MS) is a major demyelinating disease and causes sensory and motor paralysis (Compston and Coles, 2008; Reich et al., 2018). White matter degeneration in MS has been observed by magnetic resonance imaging and confirmed by histological analysis of postmortem brains (Wolswijk, 2000; Polman et al., 2005; Fisniku et al., 2008). Morphological three dimensional analysis using immunohistochemistry and confocal microscopy showed that oligodendrocytes in chronic lesions extended multiple processes and contacted demyelinated axons but failed to remyelinate those axons (Chang et al., 2002). It has been well established that myelin sheathes produced by oligodendrocytes upon remyelination are thinner and shorter (Lassmann et al., 1997). However, heterogeneity of MS lesions has also been discussed and defined based on the loss of myelin protein, an extension of plaques, the patterns of oligodendrocyte disruption, and the immunopathological viewpoint (Lucchinetti et al., 1996; Lucchinetti et al., 2000). Therefore, pathological heterogeneity is thought to be involved in the specific different mechanisms of MS. Recent studies using single-cell RNA sequence analysis reported that human oligodendrocyte heterogeneity was altered in patients with MS (Jäkel et al., 2019) and some subtypes of oligodendrocytes appeared only in the brain in Alzheimer’s disease (Grubman et al., 2019; Kenigsbuch et al., 2022). However, it is still challenging to visualize individual oligodendrocyte’s detailed morphology, including the ultrastructural level in human tissues. Future morphological studies of individual oligodendrocytes would require sophisticated technique approaches to human tissues, such as light microscopic techniques involving cellular labeling and tissue clearing and 3D-EM, in order to understand the morphological heterogeneity of oligodendrocytes in MS in human (Hama et al., 2015; Tainaka et al., 2018; Tanaka et al., 2021).

On the other hand, the current basic research visualizes the morphology of oligodendrocytes in conditions of demyelination and remyelination and has provided some interesting findings in demyelinating disease models. The generation of new oligodendrocytes and remyelination of previously myelinated axons can be reliably established following focal loss of myelin using temporally and spatially controlled demyelination (Snaidero et al., 2020). In contrast, it was recently reported that oligodendrocytes that survived after cellular injury are involved in remyelination in the cortex (Bacmeister et al., 2020). In a demyelination model of zebrafish, such surviving oligodendrocytes were observed to have morphological abnormalities and wrap myelin sheaths around neuronal cell bodies (Neely et al., 2022). These findings suggest that oligodendrocyte morphology significantly depends on the causes and states of demyelination and brain region. The heterogeneity of oligodendrocyte morphology may be higher under these pathological conditions.

It has been reported that the cell density, the volume of the nucleus, and the volume density of mitochondria in oligodendrocytes decreased in patients with schizophrenia and mood disorders (Uranova et al., 2001; Uranova et al., 2004; Birey et al., 2017). Downregulation of oligodendrocyte and myelin-related genes was observed in the brains of patients with schizophrenia and bipolar disorder compared to brains in the control group (Tkachev et al., 2003). A particular type of chromosomal translocation, t (1; 11), increases risk of major mental illness. Using diffusion MRI and iPSC-derived oligodendrocytes, Vasistha et al. revealed that t (1; 11) translocation impairs white matter integrity and modulates the morphology of oligodendrocytes (Vasistha et al., 2019). These reports attracted attention to psychiatric disorders and abnormalities of oligodendrocytes. A transgenic mouse line harboring extra copies of the myelin proteolipid protein 1 (plp1) gene showed a reduction in conduction velocity, and the behavioral changes related to schizophrenia caused abnormalities in the neuron-glia interactions at the paranodal junctions in the CNS (Kagawa et al., 1994; Tanaka et al., 2009). Abnormalities in cerebral white matter regions in patients with schizophrenia and bipolar disorder have similar pathophysiological features. In contrast, abnormalities in autism spectrum disorder and depression are minor and have biological features similar to those in healthy subjects (Koshiyama et al., 2020). Mature oligodendrocytes regulate the subtle structural modifications of myelin sheaths to influence action potential conduction velocity, and such subtle changes might affect the spatial learning memory (Cullen et al., 2021). Thus, oligodendrocyte morphology and myelin plasticity may be involved in the maintenance of neural circuits. However, the correlation between psychiatric disorders and abnormalities in oligodendrocytes has been analyzed only at the transcriptomic level and pathologically. For a complete understanding of psychiatric disorders, analysis of the individual cell morphology is necessary.

Thus, it is inferred that the morphology of oligodendrocytes is involved in the pathogenesis of neurological diseases. Further morphological analysis of oligodendrocytes specific to each pathological condition is expected to lead to a deeper understanding of the pathophysiology.

## Conclusion

Myelination of oligodendrocytes is critical for basic and higher brain functions, including sensory and motor systems, learning/memory, and social behavior. However, the mechanisms of how oligodendrocytes are engaged in the diverse CNS functions are still poorly understood. Recent advancements in imaging technologies led us to a deeper understanding of the dynamic changes and heterogeneity of oligodendrocyte/myelin morphology. Some studies utilizing those approaches supported the concept that some classes of the oligodendrocyte population can selectively myelinate a certain group of axons and alter their myelin structures, thereby modulating specific neuronal circuits and brain functions. Various intrinsic and environmental factors have been proposed to regulate the oligodendrocytes’ heterogenous morphology and functions. However, it remains unclear whether morphological changes in oligodendrocytes are necessary for memory consolidation in spatial learning or visual deficits in monocularly deprived animals. In future studies, it is essential to develop approaches precisely manipulating oligodendrocyte morphology and investigate whether the prevention of morphological changes in oligodendrocytes impairs experience dependent modulation of brain functions. Structural adaptation and impairment of oligodendrocytes and myelin have been studied in animal models and human tissues with neurological disorders, where abnormal neural functions and impaired myelin regeneration are implicated. In this regard, elucidating the subtypes of oligodendrocytes vulnerable to demyelinating diseases may facilitate the development of new therapies. Further efforts to understand the structural remodeling of oligodendrocytes and their myelin in response to environmental and behavioral changes and identification of specific neural circuits myelinated by different subtypes of preexisting and newly generated oligodendrocytes would contribute to a deeper understanding of CNS functions and development of novel therapies in neurological disorders.
